# Phenolic Compounds as Markers of Wine Quality and Authenticity

**DOI:** 10.3390/foods9121785

**Published:** 2020-12-01

**Authors:** Vakarė Merkytė, Edoardo Longo, Giulia Windisch, Emanuele Boselli

**Affiliations:** 1 Faculty of Science and Technology, Free University of Bozen-Bolzano, Piazza Università 5, 39100 Bozen-Bolzano, Italy; Vakare.Merkyte@natec.unibz.it (V.M.); giulia.windisch@unibz.it (G.W.); emanuele.boselli@unibz.it (E.B.); 2 Oenolab, NOI Techpark South Tyrol, Via A. Volta 13B, 39100 Bozen-Bolzano, Italy

**Keywords:** phenolic compounds, chemical markers, wine authenticity, wine quality, food traceability, chemometrics

## Abstract

Targeted and untargeted determinations are being currently applied to different classes of natural phenolics to develop an integrated approach aimed at ensuring compliance to regulatory prescriptions related to specific quality parameters of wine production. The regulations are particularly severe for wine and include various aspects of the viticulture practices and winemaking techniques. Nevertheless, the use of phenolic profiles for quality control is still fragmented and incomplete, even if they are a promising tool for quality evaluation. Only a few methods have been already validated and widely applied, and an integrated approach is in fact still missing because of the complex dependence of the chemical profile of wine on many viticultural and enological factors, which have not been clarified yet. For example, there is a lack of studies about the phenolic composition in relation to the wine authenticity of white and especially rosé wines. This review is a bibliographic account on the approaches based on phenolic species that have been developed for the evaluation of wine quality and frauds, from the grape varieties (of *V. vinifera* and non *vinifera*), to the geographical origin, the vintage year, the winemaking process, and wine aging. Future perspectives on the role of phenolic compounds in different wine quality aspects, which should be still exploited, are also outlined.

## 1. Introduction

Wine is a product with high commercial value and relevant cultural aspects. Its desirability on the market, combined with the high prices that consumers are willing to pay for top quality bottles, is a cause for food frauds [1,2], also with very recent examples [3]. Recently, over one million liters of counterfeit wine were discovered by the European Anti-Fraud Office [4]. Mislabeling of variety, geographical origin, or vintage year and adulteration with ethanol, sugar, and colorants are typical examples of frauds related to wine [5]. Therefore, the wine industry and consumers are highly concerned about the quality and authenticity of wine [6].

Wine quality is determined by several factors such as the type (or blend) of grape varieties, the *terroir*, the viticultural practices, the winemaking techniques, and the aging conditions [7,8,9]. The variety of grapes is a key factor in determining the wine flavor, especially during the production of premium wines. Thus, the adulteration of these types of wines with cheaper grape varieties is common [10,11,12]. *Terroir* is a French term that defines the very specific combination of geographical, climatic, and pedological factors, characterizing the growth and quality of the grapes. *Terroir* is mainly influenced by the climate and soil conditions, and it is strongly related to viticultural practices and vintage year [13,14,15].

One of the aspects related to wine authenticity is based on the (blend of) grape varieties used in winemaking, their geographical origin, and vintage. The authenticity related to the *terroir* is guaranteed by strict guidelines adopted by the European Union also based on national rules and the indications of The International Organization of Vine and Wine [16].

Wine quality evaluation is based on sensory and chemical analyses. In the sensory tasting, wine quality indicators, such as color, mouthfeel, and taste are largely, but not exclusively, influenced by the phenolic profile. Thus, phenolic compounds are widely used for the wine quality and authenticity assessment [17,18,19].

The aim of this paper is to present an overview of the current knowledge on the phenolic compounds used as chemical markers for specific winemaking practices and the assessment of wine quality and authenticity. In contrast with recent reviews on phenolic markers, this review is focusing on chemical compounds rather than analytical techniques that have been applied and statistical approaches used to process the analytical data [20,21]. The main reported aspects are related to the grape varieties (of *V. vinifera* and non *vinifera*), the geographical origin, the vintage year, the winemaking process, and wine aging.

## 2. Classification of Phenolic Compounds of Wine

Phenolic compounds are secondary metabolites present in grapes and wine that can be formed and transformed during the winemaking process. Phenolics can be classified as flavonoids (e.g., anthocyanins, flavan-3-ols, flavonols) and non-flavonoids (e.g., phenolic acids, stilbenes) [22,23]. The phenolic compounds most usually applied for the quality and authenticity assessment of wine are phenolic acids, flavonoids, tannins, and stilbenes. The role of each class of phenolics will be discussed.

### 2.1. Phenolic Acids

**Figure 1:**
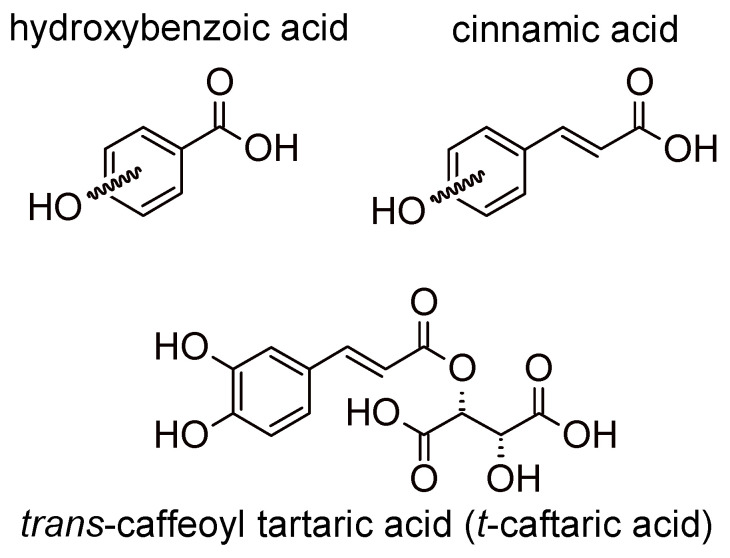
Model structures for common natural hydroxybenzoic acids, cinnamic acids, and an example of a derivative present in grape and in wine. Hydroxylation substitutions on aromatic rings are indicatively shown by the curled bonds.

There are two main groups of phenolic acids that are used for the quality and authenticity assessment and are significant for white grapes and wines: hydroxybenzoic acids (containing seven carbon atoms) and hydroxycinnamic acids (nine carbon atoms). Model structures are reported in Figure 1.

Cinnamic acids (e.g., caffeic, coumaric, ferulic, and sinapic) can be found also in two isomeric forms (*cis* and *trans*) because of the presence of a double bond. Hydroxybenzoic and hydroxycinnamic acids do not only occur in their free forms but as derivatives in conjugated or esterified forms as well. For example, hydroxycinnamic acids in wine originate during fermentation from the hydrolysis of hydroxycinnamic tartaric esters [13,24]. They can be an oxidation substrate and precursors of browning of white wines and give a bitter flavor [13,25].

### 2.2. Flavonoids

**Figure 2:**
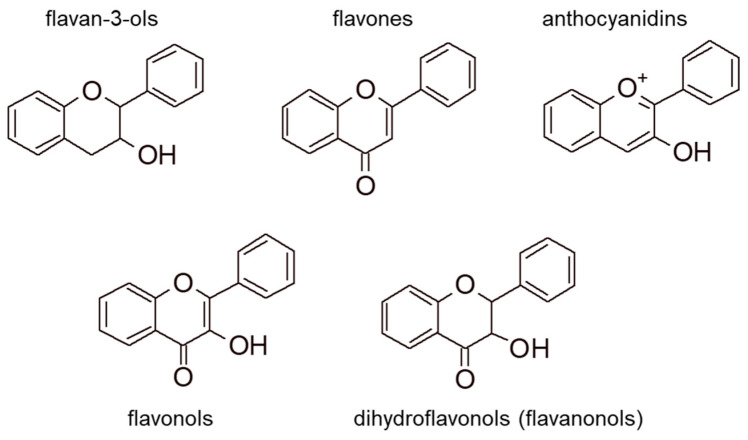
Model structures for common natural flavonoids. Hydroxylation substitutions and stereogenic configuration patterns are not explicitly shown for brevity.

Flavonoids are 15-carbon compounds including two aromatic rings bound through a three-carbon chain. Model general structures for the most common families of natural flavonoids are reported in Figure 2.

**Figure 3:**
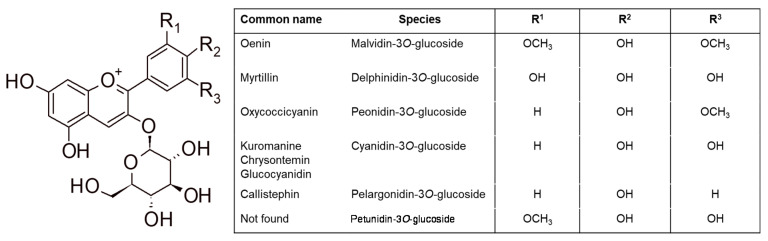
Structures of mono-glycosylated anthocyanins.

Wine flavonoids occur both in free and conjugated forms, as for example, glucosides. The most important classes of flavonoids that have been applied as chemical markers are anthocyanins, flavonols, and flavan-3-ols. The most common mono-glycosylated anthocyanin forms are summarized in Figure 3. Anthocyanins can be classified into mono-, disubstituted and trisubstituted congeners according to the total number of hydroxyl and methoxy groups present in the lateral ring (they can be 2 or 3 considering R^1^, R^2^, and R^3^ in Figure 3). Anthocyanins are not only found in simple mono-glycosylated forms, but they can also be esterified on the glycosidic moiety, such as acetyl-glucosides, *p*-coumaroyl-glucosides, and caffeoyl-glucosides (acylated anthocyanins).

Each grape variety presents a typical anthocyanin pattern [26,27]; thus, anthocyanins are the most applied flavonoids for the assessment of authenticity and quality of red wines. These protocols are based on the differentiation between (a) different anthocyanidin congeners, (b) anthocyanins mono- and di-glucosides (3-*O*-glucoside derivatives are shown in Figure 3), and (c) acylated or non-acylated anthocyanins, as reported in the section Grape variety of red wine.

Flavonols show an unsaturation between C_2_ and C_3_. They are hydroxylated in position C_3_ and have a carbonyl group in C_4_. Flavonols are present in wine as aglycones and glycosylated forms [28]. The most abundant flavonols in wines are quercetin and myricetin [29]. Differently from flavonols, flavan-3-ols have a saturated carbon bond between C_2_ and C_3_ and no carbonyl group. Catechin, epicatechin, and their derivatives (e.g., gallocatechin, epicatechin gallate) are the most abundant flavan-3-ols in wine. Some derivatives show also the presence of an ester with gallic acid at C_3_ [30]. A particular type of flavonoids, condensed tannins (or proanthocyanidins), are oligomeric or polymeric forms of flavan-3-ols and will be discussed separately.

### 2.3. Tannins

Tannins are divided into two very different chemical classes: hydrolyzable and condensed tannins; they give the astringency perception to wines [31]. Depending in which acid they are converted upon hydrolysis, hydrolysable tannins are defined as gallotannins (hydrolyzing into gallic acid) or ellagitannins (hydrolyzing into ellagic acid) [32].

**Figure 4:**
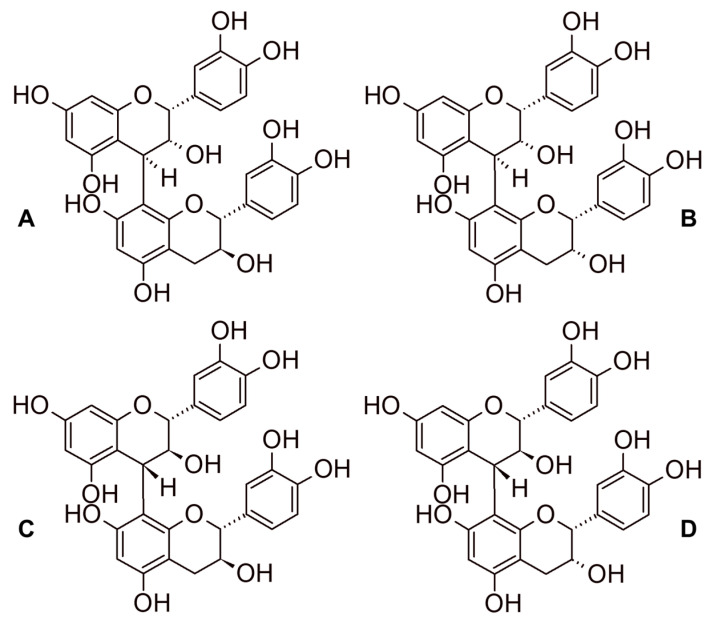
Example of isomerism for dimeric procyanidins: procyanidin B1 (**A**), procyanidin B2 (**B**), procyanidin B3 (**C**), procyanidin B4 (**D**).

Unlike hydrolysable tannins, condensed tannins can be oligomers or polymers of flavan-3-ols, depending on their degree of polymerization. Condensed tannins are also called proanthocyanidins and their degree of polymerization (DP) may range from 2 to about 20 in wine, and their solubility tends to decrease with the increasing number of monomeric units [33,34]. They show a great variability of isomers, depending on the geometry of their bindings and the type of monomers involved (Figure 4). They influence the taste (bitterness), astringencyin wine [35] and color stabilization in red wines by combining with anthocyanins [36].

### 2.4. Stilbenes

**Figure 5:**
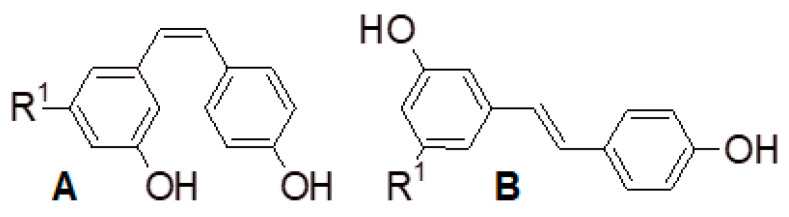
*cis*-piceid (**A**) and *trans*-piceid (**B**), when R^1^ = O-glucose; and *cis*-resveratrol (**A**) and *trans*-resveratrol (**B**), when R^1^ = OH.

The main stilbenes present in grapes and wine are resveratrol and piceid (resveratrol glucosides) in *cis* and *trans* isomeric forms (Figure 5). Stilbenes have proved to be good discriminants of the grape variety [10,23,37,38,39], grape species [5] and *terroir* [7,8,13,40] in white, rosé, and red wines.

## 3. Applications

### 3.1. Grape Variety

#### 3.1.1. Grape Variety of White and Rosè Wine

The comparison of phenolic profiles of different grape cultivars is the most studied application regarding phenolics as chemical markers and is approached using combined analytical techniques such as liquid chromatography, mass spectrometry, nuclear magnetic resonance (NMR), ultraviolet–visible spectroscopy (UV-Vis), and vibrational spectroscopy [41]. Hydroxycinnamic and hydroxybenzoic acids play an important role in the differentiation of wines made from white grapes, as it was shown (Table 1) in the studies on Czech wines [42] and on Romanian and French wines [43]. The most abundant phenolic acids in wines made from local Czech grapes were protocatechuic acid (Aurelius), gallic acid (Moravian Muscat), hydroxybenzoic acids and caftaric acid (Malverina), and *p*-coutaric and caftaric acids (Hibernal). However, the differences were not significant and could have been affected by the winemaking technology. Lampir et al. [42] applied canonical variate analysis (CVA) and showed that hydroxycinnamic acids, (+)-catechin and (−)-epicatechin were the best discriminants and were able to classify all varietal white wines 100% correctly. Magdas et al. [43] used the content of hydroxycinnamic acids in a combination with shikimic acid and (−)-epicatechin (measured by NMR) for the classification of Chardonnay, Riesling, and Sauvignon Blanc. The results using these particular groups of markers were not affected by the different geographical origins (various regions of France and Romania). Other differentiations of white wines were based on the specific set of phenolics from different classes [10] and in a combination of amino and organic acids [44], shikimic acid, caftaric acid, 2,3-butanediol, glycerol and ethanol [45], 2-*S*-glutathionylcaftaric acid, and sensory perception [46].

A major study on white, rosé, and red Apulian wines and their blends has been done by Ragusa et al. [23]. Most rosé wines were composed of Negroamaro grapes and yielded similar concentrations of gallic and syringic acids. All rosé wines showed higher values of *trans*-resveratrol in comparison with white wines. Baron et al. [37] focused on the phenolic components of 48 rosé wines from the Czech Republic. Anthocyanins and caftaric acid were the most abundant phenolic compounds in different grape varieties. Wines made from Blaufränkisch grapes were rich of hydroxycinnamic acids, while Pinot Noir and Zweigeltrebe wines had distinctive profiles (very high and low concentrations, respectively) of catechin, epicatechin, and both isomeric forms of resveratrol. In both [23,37], a set of various phenolics was selected for the possible discrimination of these wines.

#### 3.1.2. Grape Variety of Red Wine

For the quality control of red grape varieties, the profile of anthocyanins is typically used. These compounds are presented in Table 2. Red grape cultivars of *Vitis vinifera* contain a specific profile (relative proportions) of (mostly) monoglucosylated anthocyanins, whereas other *Vitis* species (so-called American varieties, not admitted to produce wine marketed in the EU) contain also diglucosylated anthocyanins, which are discussed in the next chapter.

Cejudo-Bastante et al. [22] have analyzed Tempranillo, Tortosi, Bobal, Moravia Agria, Moravia Dulce, and Rojal wines. The observed anthocyanins were free, acetyl-, coumaryl, and caffeoyl-esters of cyanidin-3-*O*-glucoside, delphinidin-3-*O*-glucoside, malvidin-3-*O*-glucoside, peonidin-3-*O*-glucoside, and petunidin-3-*O*-glucoside. In Tempranillo wines, the concentrations of 3-*O*-glucosides were distributed as follows:

(1) glucosides: Mv-3-glu > Pt-3-glu > Dp-3-glu > Pn-3-glu > Cy-3-glu;
(2) 3-acetyl-glucosides: Mv-3-ace > Pt-3-ace > Pn-3-ace > Dp-3-ace > Cy-3-ace;
(3) 3-*p*-coumaryl-glucosides: *trans*-Mv-3-cum > Dp-3-cum > Pn-3-cum > *cis*-Mv-3-cum > Cy-3-cum;
(4) caffeoyl-3-glucosides: Mv-caf > Pn-caf.
  (Mv = malvidin; Pt = petunidin; Dp = delphinidin; Pn = peonidin; Cy = cyanidin)

Pt-3-cum was not present. In comparison to Tempranillo wines, the concentration of Mv-3-glu in Tortosi wines was twice lower and Cy-3-glu, Pt-3-ace, Pn-3-ace, Dp-3-cum, and Pn-caf were not present. Conversely, Bobal wines had rather low concentrations of all anthocyanins, but only Pt-3-ace, Pn-3-ace, and Pn-caf were not identified. The only anthocyanins that were present in Moravia Agria wines were Cy-3-ace, Mv-3-ace, *cis*-Mv-3-cum, Pn-3-cum, *trans*-Mv-3-cum, and Mv-caf; in Moravia Dulce: Dp-3-glu, Pt-3-glu, Pn-3-glu, Mv-3-glu, Cy-3-ace, Mv-3-ace, *cis*-Mv-3-cum, Pn-3-cum, and *trans*-Mv-3-cum; in Rojal: Pn-3-glu, Mv-3-glu, and Cy-3-ace.

The absence of a specific anthocyanin in grapes can be exploited for the authenticity assessment. La Notte et al. [47] and Storchi et al. [48] noticed that acetylated anthocyanins and Mv-caf were not present or present in traces in Sangiovese grapes and wines. Instead, these grapes had very low concentrations of 3-*p*-coumaryl-glucosides that were very abundant in Malbec and Syrah grapes.

Pinot Noir, Pinot Meunier, and Pinot Madeleine grapes do not synthesize acetylated anthocyanins, as well [49].

Revilla et al. [27] have studied anthocyanins in Cabernet Sauvignon, Tempranillo, Merlot, Garnacha, Graciano, and Mencia wines. The most abundant compounds in Cabernet Sauvignon were Mv-3-glu, Mv-3-ace, Mv-3-cum, Dp-3-glu, Pt-3-glu, and Pn-3-glu. Interestingly, in Tempranillo samples, the content of 3-*O*-glucosides (except Mv-3-glu) was twice or higher than in Cabernet Sauvignon. Mv-3-glu > Mv-3-ace > Pn-3-glu > Mv-3-cum > Dp-3-glu > Pt-3-glu were the most abundant anthocyanins in Merlot. Merlot had the highest concentrations of Cy-3-glu (> Pn-3-glu > Pn-3-cum) of all studied wines. Instead, Garnacha showed the highest concentration of Mv-3-glu and low concentrations of other anthocyanins. Finally, in Garciano wines the distribution of anthocyanins was: Mv-3-glu > Pn-3-ace > Mv-3-cum > Pt-3-glu > Dp-3-glu. Gonzalez-Neves et al. [50] were mainly focused on the content of 3-*O*-glucosides in Merlot, Syrah, and Tannat wines. Merlot samples showed the same anthocyanins pattern as previously discussed. It also had the highest content of total acetyl glucosides among all wines. Both in Syrah and Tannat, the most abundant compound was Mv-3-glu and the least abundant was Cy-3-glu. The pattern of 3-*O*-glucosides in Syrah were Pn-3-glu > Pt-3-glu > Dp-3-glu and in Tannat Pt-3-glu > Dp-3-glu > Pn-3-glu. Garcia-Beneytez et al. [51] have shown that Dp-3-ace, Cy-3-ace, and Pt-3-ace were present only in Cabernet Sauvignon, Merlot, and Monastrell wines and were not found in Alicante Bouschet, Bobal, Garnacha, and Tempranillo. Teinturier varieties (including Alicante Bouschet, Morrastel Bouschet and Petit Bouschet) contain peonidin 3-*O*-glucoside (as the major anthocyan) not only in skin cells but also in the flesh according to the same study.

Muccillo et al. [52] analyzed the trend of the malvidin-3-*O*-glucoside (Mv-3-glu), 3-acetylglucoside (Mv-3-ace), and 3-*p*-coumarylglucoside (Mv-3-cum) in studied red wines. In Piedirosso, Cabernet Sauvignon, Merlot, and Lingua di Femmina wines, the distributions according to the concentrations were Mv-3-glu > Mv-3-ace > Mv-3-cum and differed from Aglianico wines, where anthocyanins were distributed as Mv-3-glu > Mv-3-cum > Mv-3-ace. This observation came with an agreement of previous studies related with the anthocyanin content of these wines. Kumšta et al. [53] have found that a combination of delphinidin-3-*O*-glucoside, malvidin-3-*O*-glucoside, peonidin-3-*O*-coumarylglucoside, and delphinidin-3-*O*-acetylglucoside can cluster wines made from Blaufränkisch, Blauer Portugieser, and Saint Laurent grapes without influence of the geographical origin. Furthermore, the highest concentrations of malvidin derivatives were found in Blauer Portugieser, while Blaufränkisch was noted to the markedly lower content of acylated anthocyanins than glycosidic ones.

Moreover, some important studies on the anthocyanin profiles of grape skins concerning Nebbiolo and Cabernet Sauvignon varieties were done [54,55]. Both investigations agreed that the most abundant anthocyanin in Nebbiolo grape skins was peonidin-3-*O*-glucoside. The percentage between disubstituted anthocyanins and Pn-3-glu was affected by growing location [55]. The loss of disubstituted anthocyanins during the skin maceration was observed in both Nebbiolo and Cabernet Sauvignon (lesser than in Nebbiolo) grapes, as well as the increment of trisubstituted anthocyanins [55]. During the vinification, Pn-3-glu tends to oxidize, and its concentration decreases. However, Pn-3-glu is the second most present anthocyanin in Nebbiolo wine after Mv-3-glu [56].

Pisano et al. [14] have noticed that malvidin-3-*O*-glucoside, malvidin-3-(6-*O*-acetylglucoside), and malvidin-3-*O*-glucoside-4-vinylguaiacol (or malvidin-3-(6-*O*-p-coumaroylglucoside) are good discriminants between Aspiran, Bonarda, Cabernet Sauvignon, Malbec, Merlot, Sangiovese, Syrah, and Tempranillo wines from Argentina. A major study of 11 single-cultivar Italian red wines was performed [57] and possible chemical markers for seven varieties were found: hydroxycinnamates for Cannonau wines; anthocyanins—Teroldego; flavan-3-ols—Aglianico, Nerello, Nebbiolo; flavan-3-ols and flavonols—Sangiovese; amino acids and metabolites that contain nitrogen—Primitivo. In addition, ratios between specific anthocyanins [58,59] or their combination with other secondary metabolites [60] were applied. Using a set of anthocyanins, their proportions and phenolic and organic acids abundances were used to build a principal component analysis (PCA) model, in which the studied grape varieties (Cabernet Sauvignon, Feteasca Neagra, Mamaia, and Merlot) were 100% classified correctly, except for Pinot Noir, which was classified only in 87.50% of cases, since the phenolic profiles of Pinot Noir and Feteasca Neagra were quite similar [58]. The study [59] emphasized that both Corvina Veronese and Negro Amaro wines have high levels of disubstituted compounds and low levels of acyl derivatives. These wines can be chemically distinguished from each other with laricitrin, syringetin (that are low in Corvina Veronese), quercetin, and kaempferol (that are high in Negro Amaro). The anthocyanic profile of Primitivo showed high contents of (3′,4′,5′) trisubstituted flavonoids, such as laricitrin, myricetin, and syringetin, as well as a high content of trisubstituted anthocyanins, such as petunidin and malvidin derivatives and a lower content of isorhamnetin and kaempferol derivatives. De Rosso et al. [60] presented the application of indexes of laricitrin, delphinidin, and petunidin for the detection of Primitivo and Negro Amaro in the wine blends.

In addition, other researchers suggested combining the profile of anthocyanins with the content of phenolic acids [61], flavonols [18], phenolic acids, and flavan-3-ols [62]. Based on the statistical evaluation, flavan-3-ols alone [63] or combined with phenolic acids [64] and condensed proanthocyanidins [65,66] were found to distinguish well Graciano, Tempranillo, Cabernet Sauvignon, Cabernet Franc, Carménère, Merlot Pinotage, Syrah, and Sangiovese grape varieties. Interestingly, a set of specific markers was observed for Carménère and Merlot wines produced in Chile: a ratio of total quercetin and total myricetin combined with the concentration of myricetin itself [29].

The fingerprint composed by phenolic acids, flavonoids, tannins, and stilbenes can be used for the classification on the basis of mainly local grape variety in terms of producing country, as it was recently shown for Croatian wines [19]. In this study, taxifolin and peonidin acetylglucoside were differentiating red wines (Cabernet Sauvignon, Merlot, Plavac mali, Teran), whereas *cis*-piceid was efficient in differentiating white monovarietal wines (Chardonnay, Graševina, Malvazija Istarska, Maraština, Muscat Blanc, Pošip). Martelo-Vidal et al. [38] applied chemometric methods to Rías Baixas and Ribeira Sacra wines that determined significantly high concentrations of malvin in Rías Baixas and *trans*-resveratrol in Ribeira Sacra. These phenolics, together with syringic acid, oenin, (+)-catechin, (−)-epicatechin, and quercetin, proved to be a good set for the sample discrimination. Another study was mainly focused on the local grape varieties Vranac, Kratosija, and wines from Cabernet Sauvignon grapes [67]. It showed that Vranac distinguished in high contents of anthocyanins, Kratošija—hydroxycinnamic acids and Cabernet Sauvignon—flavan-3-ols. Ragusa et al. [39] reported that Negroamaro and Primitivo can be differentiated by syringic acid, hydroxytyrosol (that are high in Primitivo), and *trans*-resveratrol (high in Negroamaro). Finally, Salvatore et al. [68] described phenolic profiles of Lambrusco di Sorbara, Lambrusco Salamino di Santa Croce, and Lambrusco Grasparossa di Castelvetro. Lambrusco di Sorbara had significant concentrations of *p*-coumaric and caffeic acids, Lambrusco Salamino di Santa Croce—gallic acid, Lambrusco Grasparossa di Castelvetro—myricetin and quercitin.

Even though some studies [1,9,32,69,70] did not identify specific phenolic markers and used spectral areas in their quality control assessment, the spectroscopic analyses showed good discrimination among wines made with both white and red grape varieties with a high sample variability. However, Magdas et al. [71] have reported a strong overlap between Chardonnay and Sauvignon samples. The final separation using a linear discriminant analysis among all four cultivars (Riesling and Pinot Gris as well) was only 84%.

A new class of macrocyclic proanthocyanidins was recently discovered in grapes and wine. The congeners of cyclic (crown) oligomeric proanthocyanidins (procyanidins and prodelphinidins) are currently under study to evaluate their suitability as potential chemical markers for white and red grape varieties [72,73,74]. The ratios of cyclic compounds *versus* the sum of cyclic and non-cyclic congeners *per* number of monomer units showed an interesting relationship with the grape variety (red or white).

In addition, new acylated flavonols were recently identified [75] in Tannat, Marselan, Syrah grapes, and wines produced in the Southern Uruguay. They are acetylated derivatives of the flavonol glucosides containing methoxylated aglycones, such as isorhamnetin, laricitrin, and syringetin, and they might be used for the quality and authenticity assessment.

#### 3.1.3. Non Vitis Vinifera Grape Species

The most cultivated grapes used to produce wines belong to the *Vitis vinifera* grape species, since the regulations of the European Union allow only this species to be used for a commercial purpose [38]. Currently, there are only a few studies dealing with the evaluation of phenolic profile of hybrids and other species than *Vitis vinifera* (Table 3). It has been noticed that the anthocyanin profile of non *Vitis vinifera* grapes consist of 3,5-diglucosidic forms [36]. Small concentrations of these compounds can be also found in some *Vitis vinifera* grapes and wines [76,77,78,79]. In 2012, the International Organization of Vine and Wine (OIV) published a list of maximum acceptable limits of various chemical compounds, including the amount of malvidin-3,5-diglucoside (max. 15 mg/L) that can be used as a chemical marker of non *Vitis vinifera* grape species [80].

The content of the anthocyanin 3,5-diglucosides proved to be a good chemical marker for the differentiation of *Vitis vinifera* red wines and Isabel (*Vitis vinifera* × *Vitis labrusca* hybrid) wines by Nixdorf et al. [81]. Isabel is a hybrid grape cultivar that covers half of Brazilian grape production, and it is used to produce table wine, grape juice, and other drinks and food products.

Common wine adulteration cases in Poland are based on labeling wines made from hybrid Rondo (*Vitis amurensis* × *Vitis vinifera*) grapes as made with the Zweigelt (*Vitis vinifera*) grapes. To provide tools to discover these frauds, Stój et al. [5] analyzed the content of phenolic acids, flavonoids, tannins, and stilbenes of wines from both grape varieties. The 3,5-diglucosidic anthocyanins, Rondo wines had higher amounts of gallic acid and stilbenes (*trans*-piceid and *cis*-piceid). Spine grape (*Vitis davidii Foex*) is one of the main wild grape species growing in East Asia, and it is used mainly for red and white wine production [82]. The wine made from spine grapes contains malvidin-3,5-diglucoside, syringetin-3-*O*-glucoside, dihydroquercetin-3-hexoside, and coutaric acid [82]. Native Chinese species, such as *Vitis amurensis* and *Vitis davidii* were studied by Li et al. [83]. Wines made from *Vitis davidii* grapes were also characterized by higher concentrations of hydroxycinnamic acids than hydroxybenzoic acids and distinguished by the high contents of kaempferol-3-*O*-glucoside and quercetin-3-*O*-rhamnoside, in comparison with other grape species. Burns et al. [84] used the proportion of mono-glucoside and acetylated anthocyanins to distinguish Cabernet Sauvignon wines from the hybrid grape varieties from the North America: Baco, Seybel, Clinton, Jacquez, and Othello. Gougeon et al. [85] investigated the authenticity (origin and vintage) of Cabernet Sauvignon (*Vitis vinifera*) and Beihong (*Vitis vinifera* and *Vitis amurensis*). Using the content of various phenolic compounds (mainly phenolic acids), good separations between samples were observed in terms of grape variety and *terroir*. Unfortunately, wines were not clustered according to the vintage.

### 3.2. Geographical Origin and Phenolic Compounds

The mineral content and the stable isotope ratios (that are present in soil) are a function of geographical origin of wines and are able to describe plant growth and development, environmental contamination, and geological factors [86]. Nevertheless, also phenolic compounds were applied for the evaluation of the geographical origin; in fact, the composition of phenolic compounds in wine does not only depend on the grape variety, but it is also influenced by the viticulture practices, environmental conditions, and winemaking technologies [87,88]. In addition, the origin is an important factor for the consumer’s choice, as well as for the protection of the reputation and value of products that are recognized by the European Union under PDO (Protected Designation of Origin) and PGI (Protected Geographical Indication) classifications [15,89].

Some studies were done on the evaluation of the phenolic content (hydroxybenzoic and hydroxycinnamic acids, flavan-3-ols and stilbenes) of Riesling wines from the Czech Republic [8,13,87]. Using a CVA, significant markers of the geographical origin of Riesling wines proved to be *p*-coutaric acid, (+)-catechin, (-)-epicatechin, *trans*-resveratrol, and *cis*-resveratrol (Table 3). To emphasize their suitability as markers for the geographical origin, different vintages of Riesling wines were examined, and the analysis showed that these phenolic compounds were not influenced by the vintage year [8]. As already mentioned above, another study of white wines made from European and interspecific grape varieties from Czech Republic highlighted the importance of hydroxycinnamates (caftaric acid), flavan-3-ols ((+)-catechin, (–)-epicatechin), and stilbenes (*cis*-piceid), in addition to hydroxybenzoic acids (protocatechuic acid and *p*-hydroxybenzoic acid) for the differentiation of their geographical origin [40]. Phenolic acids were shown to be important markers for the separation of Chardonnay, Feteasca Regala, Pinot Gris, Riesling, and Sauvignon Blanc wines from different geographical regions of France and Romania [43,69,70,71] and for Australian Chardonnay and Cabernet Sauvignon wines [88]. Interestingly, the differentiation among wines that came from the same country was not possible using a surface-enhanced Raman spectroscopy (SERS) [69], but it was achieved with a FT-Raman [70] having the same samples (as in the study with SERS) and proved to be a sufficient method for other white wine analysis [43]. In the case of Chardonnay wines from Australia, France, Israel, and various regions of Italy, flavonoids appeared to show the highest discrimination potential [89]. Furthermore, using these chemical markers, it was possible to separate wines according to their aging technique: barrels vs. stainless steel tanks [71]. Recently, a comprehensive metabolomic workflow has been applied to discriminate the geographical origin of several Italian monovarietal red wines in their different *terroirs* [57]. Several putative biomarkers of origin were identified (e.g., flavan-3-ols for Aglianico, Sangiovese, Nerello, and Nebbiolo, flavonols for Sangiovese, and hydroxycinnamates for Cannonau.

There is a lack of scientific literature regarding the geographical authenticity of rosé wines. Lambert et al. [90] have done an extensive study of the quantification of 152 phenolic compounds in rosé wines from different European countries. This research was focused on the establishment of typologies of worldwide rosé wines. However, statistically targeted studies are needed to check which of the phenolic compounds are the most suitable as chemical markers.

Several phenolic compounds were found to represent well the *terroir* conditions of red wines. For example, flavonols were applied for various wines from France and Spain [28], phenolic acids and flavan-3-ols were applied for Cabernet Sauvignon, Cabernet Franc, Carménère, Merlot, and Syrah cultivated in China [12,66], catechin and quercetin were applied for Cabernet Sauvignon wines from Balkan regions [91] and catechin was applied for Syrah wines from Brazil [11]. However, the correct differentiation of wines according to their geographical origin, in all previously mentioned studies, ranged between 73 and 89%. The profile of anthocyanins showed to be a good marker of Merlot wines from neighboring countries in the South America in combination with a support vector machine (SVM) classifier [6]. High accuracy in prediction (93.73%) was obtained in the distinction between Brazil versus non-Brazilian wines, while the least accurate model was 79.16% (for the Merlot from Chile versus non-Chile). Furthermore, anthocyanins were used for Cabernet Sauvignon and Merlot wines produced in China and Balkan Peninsula, red wines from Argentina and Czech Republic. The main chemical markers for the authenticity in these studies were malvidin-3-*O*-glucoside and its derivatives [14,19,92,93] as well as delphinidin-3-*O*-glucoside and delphinidin-3-*O*-*p*-coumarylglucoside [53]. Likewise, anthocyanins measured with the UV-Vis spectroscopy were applied for the recognition of monovarietal and blended wines from different viticulture regions of Spain [94]. Regarding Malbec wines from Argentina, the evaluation of their phenolic profiles showed that the most descriptive compounds of the *terroir* were caftaric acid, quercetin-3-*O*-glucoside, and (+)-catechin [15]. Spanish wines Rías Baixas and Ribeira Sacra labeled with Designation of Origin, along with various German, Greek, Croatian wines, were determined using phenolic acids, flavonoids, tannins, and stilbenes, as reported in Table 4 [7,38,45].

### 3.3. Winemaking

All the steps of the winemaking procedure (prefermentation treatments, fermentation/maceration, stabilization, addition of fining agents, corrections, and aging) may impact the quality of wines. The higher fermentation and maceration temperatures increase the content of phenolic compound; the extended maceration time increases the levels of tannins [95].

Concerning maceration and fermentation, there are only some studies based on the associated phenolic quality markers. The phenolic profile of Cava sparkling wine was characterized by Bosch-Fusté et al. and Izquierdo-Llopart et al. [96,97]. The quality parameters were phenolic acids (Table 5). PCA showed a good clustering and separation of monovarietal and polyvarietal wines using syringic, gallic, caffeic, coumaric, and caftaric acids. These analyses confirmed that hydroxycinnamic acids, their esters, and tartaric acid are important markers in sparkling wines. In addition, rosé and Chardonnay cavas had the highest phenolic contents, while the classical blends of Macabeu, Xarel·lo, and Parellada cavas contained low levels [97].

Dupas de Matos et al. [98] presented differences in the phenolic composition of Pinot Blanc wines made with and without prefermentative cold maceration in the presence of pectolytic enzymes. The samples from both winemaking procedures were best described by the concentrations of *trans*-caftaric acid and astilbin.

Suriano et al. [99] investigated the maceration held with different amounts of destemmed Primitivo grapes. Anthocyanins were the compounds that were most differentiating between the maceration without and with stems. Primitivo wines made with destemmed grapes had higher concentrations of monomeric and total anthocyanins both at racking and after 12 months storage compared to the wines with 25% and 50% grapes with stems that were richer in tannins. Anthocyanins were used as chemical markers of three types of maceration of Merlot, Syrah, and Tannat [50]. Using the relative anthocyanin profile, it was possible to cluster the samples according to the winemaking technique. In addition, the content of anthocyanins increased during cold soaking maceration in Merlot and Tannat wines. Merkytė et al. [100] compared different winemaking processes of Pinot Noir wines and reported that cyclic and non-cyclic proanthocyanidins and their relative ratio showed to be affected by the use of raisin grapes for winemaking and by the occurrence of stuck fermentations. The ratios between cyclic proanthocyanidins and total proanthocyanidins (with the same number of monomers) were the highest in wine that experienced stuck fermentation in comparison with other samples, possibly because they may be extracted in the first steps of maceration. The wine from raisin grapes showed the lowest ratios, meaning that it contained higher concentrations of cyclic proanthocyanidins among all the samples.

Red wine blending was studied by Lorenzo et al. [101], who evaluated the differentiation of wines according to the aging time and the type of wine using discriminant analysis, showing that the main markers were anthocyanins and their derivatives.

Cejudo-Bastante et al. [22] studied the phenolic profile of different stages of vinification in grapes skins, musts, and wine in Tempranillo and minority grape varieties of Castilla-La Mancha region (Spain), showing that rosé wines with the highest amount of flavonols were Tortosí and Bobal wines, while Moravia Dulce and Moravia Agria wines showed low quantities. The highest values of anthocyanins were observed in Bobal and Moravia Agria wines (in the middle and at the end of alcoholic fermentation). However, their content decreased after malolactic fermentation. Interestingly, Tortosí and Moravia Dulce wines showed increment of anthocyanins after malolactic fermentation. In addition, wines made from Rojal grapes were practically absent both of flavonols and anthocyanins. Another qualitative study was done by Loizzo et al. [102] that was focused on an investigation of Passito wines made from non-macerated white grapes: Guarnaccia, Malvasia, and Moscato. Phenolic compounds found in the highest concentrations were gallic and caftaric acids, (+)-catechin and oligomeric procyanidins (due to the polymerization of simple flavonoids).

### 3.4. Aging

During the wine aging in wooden barrels, phenolics migrate from wood to wine and cause changes in color and mouthfeel sensations. Moreover, the micro-oxygenation through the pores of wood is influencing the color of red wines (decrement of free anthocyanins and formation of polymeric pigments). The profile of phenolic compounds in the aged wines depends on many factors, such as type of barrel wood, environmental humidity, toasting degree, wine alcohol content, aging time, wood structure, and its polyphenolic load. The typical taste of the wines stored in wood is one of the quality parameters [32,103]. The chemical changes of wine during aging can be observed in bottles. The study by Arapitsas et al. [104] was focused on the interactions between SO_2_ and phenolics, as well as amino acids in wines produced from 1986 until 2016. It showed that the best discriminants for white wines were sulfonated indoles, and for red wines, they were sulfonated monomeric and oligomeric flavan-3-ols.

A few studies suggested that phenolic acids diffusing from wood can be used as chemical markers for the wines aged in wooden barrels (Table 6). Matejicek et al. [103] found that the content of ellagic acid showed a noticeable difference between wines that were aged in the medium and highly toasted barrels; thus, ellagic acid can be used as the marker of maturity of oak barrique wines. However, the degree of toasting in each toasting category did not affect its concentration. Another investigation by Sanz et al. [105] concerning phenolic acids showed that 2,4-dihydroxybenzoic acid together with certain flavonoids (e.g., robinetin, fustin, butin, tetrahydroxydihydroflavonol, and trihydroxymethoxydihydroflavonol) were a good set of discriminators of wines aged in acacia wood, since these compounds were not present in wines stored in oak wood. Alañón et al. [106] showed that the profiles of benzoic acids (protocatechuic, gallic, and ellagic acid) and monomeric anthocyanins were the marker for the wine aged in chestnut wood. The content of protocatechuic acid was different in wine made in non-toasted and toasted barrels, but its concentration was not changing during the aging (in 3 and 6 months), while the content of gallic and ellagic acids were discriminants of wine aging. The monomeric anthocyanins described the best changes during aging in non-toasted chestnut barrels. In addition, the lower concentration of total flavonols in wines that were treated with chestnut chips than the samples in barrels might be a possible marker for this type of wine aging. Chinnici et al. [107] showed that flavonoids such as eryodictiol, sakuranetin, pinocembrin, and chrysin appeared to be the most distinctive phenolic compounds of wine stored in cherry wood. These compounds were not found in samples aged in oak barrels or steel tanks.

In the studies of wine aging comparing the storage in oak barrels *versus* the addition of oak chips [108,109], protocatechuic and vanillic acids, together with (-)-epicatechin and anthocyanins were the most discriminant markers. Del Alamo et al. [108] investigated the red wine aging in different oak wood (American, French, and Hungarian) systems: barrels, chips, and staves. They indicated the set of chemical markers; moreover, they also analyzed the same samples after 2 years of storage in bottle and found that differences between these three systems grew during the bottling period. Ortega-Heras et al. [109] showed that wines treated with chips are higher or lower in phenolic content than wines aged in barrels depending on their variety and vintage. Baiano et al. [110] investigated Aglianico and Montepulciano wines treated with and without oak chips; the usage of oak chips determined an increase of polymerized anthocyanins and tannins in Aglianico and only polymerized anthocyanins in Montepulciano wines. After one year of storage in bottles, the decrease in monomeric anthocyanins concentration was higher in samples treated with oak chips in both type of wines. Finally, extensive studies of wine aging in barrels made from different type of wood (French oak, American oak, acacia, and chestnut) were carried out by Basalekou et al. [32,111]. The first studies suggested that the Fourier-transform infrared spectroscopy (FT-IR) spectral regions from 1800 to 1500 cm^−1^ and from 1300 to 900 cm^−1^ showed a good discrimination of differently aged white and red wines. However, the correlation based on their aging time was poor. In the later publication, they focused on the content of ellagitannins. The levels of ellagitannins depended on the type of wood, its geographic origin, and the wine aging time. The wines aged in chestnut wood had the highest concentration of ellagic acid, whereas French oak appeared to enhance the content of ellagic acid more than American oak. The lowest concentration of ellagic acid was observed in wines treated with chips. In agreement with previous observation, ellagic acid was absent in samples aged in acacia wood. Furthermore, the application of ellagitannins as quality markers could assist in the industry, since they can be used for both white and red wines with the same methodology.

### 3.5. Vintage Year

The vintage year is an important quality parameter, that strongly influences the price of wine, and it is mainly affected by various environmental factors such as the viticultural practice and meteorological (climatic) conditions. During the wine aging in bottle, the phenolic composition changes and affects the wine color, astringency, and bitterness perceptions [17,112].

Some studies [7,44,45] investigated the phenolic profile of white wines over two different vintages using the NMR technique. PCA and partial least squares-discriminant analysis (PLS-DA) of the NMR spectra showed a clear differentiation between the samples, using mainly phenolic acids as variables. Anastasiadi et al. [7] demonstrated that the vintage is related to the environmental conditions, since both white and red wines made in 2005 were characterized by lower concentrations of polyphenols, which can be explained by the overhydration of grape berries due to heavy rainfalls that year in Nemea (Greece). Studies [43,69,70,71] using SERS and FT-Raman analysis showed the application of phenolic acids for white wine made with grapes from different harvest years. In the studies of 2018 [69,70], a weaker classification (by linear discriminant analysis (LDA)) was obtained using SERS (83.3%) in comparison to FT-Raman (96.9%), which proved to be better analysis technique for the vintage differentiation, since SERS does not provided signals for specific phenolic acids that are important discriminators.

Other studies based on vintage analysis were made with red deep-colored wines and are presented in Table 7. In Pinotage wines, concentrations of caffeic acid were stable throughout the aging process, while levels of malvidin-3-*O*-glucoside were decreasing. It was suggested that the increment of the ratio between caffeic acid and malvidin-3-*O*-glucoside reveals the older vintages of Pinotage, as well as the formation of pinotin A [113]. In Tempranillo wines produced in 2000, the percentages of delphinidin-3-*O*-glucoside, petunidin-3-*O*-glucoside, and malvidin-3-*O*-*p*-coumarylglucoside were higher, and the content of malvidin-3-*O*-glucoside was lower than in 2001 and 2002 due to climatic factors linked to the year of production [114]. The study on Sangiovese wines showed the decrement of the absolute concentration of simple glucosides of anthocyanins and vitisin B pigments in the older wines, whereas pinotin A pigments were increasing through the years [115]. In the work of Chira et al. [17], the profile of anthocyanins was in combination with tannins and sensory data. However, the most relevant marker for the vintage year differentiation in both Cabernet Sauvignon and Merlot wines was the mean degree of polymerisation of proanthocyanidins. The study by Eder et al. [116] confirmed that the content of total monomeric anthocyanins and malvidin-3-*O*-glucoside are suitable markers to assess the vintage year. The concentrations of monomeric anthocyanins were reduced by more than 50% after two years, while pyranoanthocyanins (vitisin A, B, and pinotin A) were independent from the wine age. Monomeric and dimeric flavan-3-ols, proanthocyanidins, and total phenolic content were perfect differentiators of Cabernet Franc, Merlot, Sangiovese, and Syrah wines produced in 2006 and 2007 [65]. The concentrations of catechin, proanthocyanidins, total monomers, and total dimers were significantly different in both Cabernet Franc and Merlot wines from both vintages. The content of these compounds was higher in wines from 2006. Conversely, Sangiovese from vintage 2007 had lower concentrations of catechin, epicatechin, total monomers, and total dimers compared to wines produced during the vintage 2006. The same trend was in Syrah wines, except for the total dimers.

As discussed before, different classes of phenolic compounds can be combined to get a good set of quality markers. Bellomarino et al. [111] reported that a mix of phenolic acids, flavonoids, and stilbenes was suitable to assess the vintage year of Cabernet Sauvignon wines, since their concentrations changed during aging. The content of tartaric acid, sinapic acid, and (+)-catechin decreased, but the concentrations of vanillic acid and resveratrol increased. Instead, Li et al. [12] applied a set of flavonoids for identifying the vintages of Cabernet Sauvignon that 100% correctly separated young and old wines and showed 81% differentiation among young wines. Geana et al. [1] used the FT-IR technique and were focused on the signals in the 1600–900 cm^−1^ spectral region (phenolic acids, flavonoids, tannins, amino acids, aldehydes, sugars, alcohols, etc.) that were able to separate samples from nine different vintages. Previously, the same research team showed the successful application of the combinations of the NMR’s fingerprint, the profile of anthocyanins, and certain anthocyanin ratios (cyanidin-3-*O*-glucoside/malvidin-3-*O*-glucoside, petunidin-3-*O*-glucoside/malvidin-3-*O*-glucoside, malvidin-3-*O*-(6-*p*-coumaroyl)glucoside/malvidin-3-*O*-glucoside and total anthocyanins/malvidin-3-*O*-glucoside) to assess the vintage years from 2009 to 2014 [1].

## 4. Conclusions

Phenolics are important chemical compounds present in wines that can be used as quality and authenticity fingerprints in terms of grape variety, grape species, *terroir*, winemaking, aging conditions, and vintage. In the last two decades, the research based on the phenolic profiles of wines increased exponentially.

However, the majority of studies concerning quality control were performed on red grape varieties (mainly Cabernet Sauvignon, Merlot, Syrah, Pinot Noir, Tempranillo, and Sangiovese); thus, there are less scientific studies on white wine (mainly Chardonnay, Riesling, and Sauvignon Blanc) and especially on rosé wines. Markers applied for the differentiation according to the origin were phenolic acids for white wines and various flavonoids, especially anthocyanins for red wines. Markers for the vintage year are phenolic acids (white wines) and anthocyanins (red wines, mainly Cabernet Sauvignon). Markers for winemaking and aging in wood tanks are ellagic acid and other phenolic acids, flavonols, and tannins. To differentiate grape varieties, phenolic acids, stilbenes (white wines), and anthocyanins (red wines) were mainly used. Liquid chromatography with various detectors (e.g., diode array detection (DAD) and mass spectrometry (MS)) were the main techniques to determine each chemical marker in wine, in comparison with NMR and vibrational spectroscopy that were mostly used for the geographical origin and vintage evaluation. Concerning statistical analyses, PCA, linear discriminant analysis (LDA), PLS-DA, and analysis of variance (ANOVA) were the most applied. Some publications presented newly observed phenolic compounds that have a high potential to be used as chemical markers in the different fields of quality control. However, wine is a complex food matrix that is influenced by many factors, and not many studies were able to focus on the definition of selected chemical markers able to account for different variables (grape variety, geographical origin, winemaking, aging, and vintage). In fact, it could be argued that this currently very active research field could still present a fragmented landscape. This could be the direct result of the evident dependency of the phenolic profile on many variables at the same time. As a result, few analytical and statistical methods for assessing quality could produce a totally generalized model, but they have had to be contextualized instead for certain factors and within the extent and variety of the dataset investigated. Still, very high rates of correct classifications (based on grape variety, geographical origin, etc.) could be achieved, e.g., by optimizing and refining the applied selection of markers. Many discriminant and classification techniques reported in this review have been applied to wine samples, having phenolic compounds as the principal markers and according to the aim of the model (classification by variety, by origin, etc.). In these examples, when the methods were accordingly calibrated and applied, the performances in classification or discrimination were always very high (in general between 90% and 100%). However, one consequence of the unexplained underlying complexity is that the great part of these studies has produced a set/sets of relationships between only specific phenolic compounds or classes, and specific factors, excluding (necessarily) all others; hence, on one side, these models were only validated within the limits of the studies themselves, so representing just a portion of the whole complexity; on the other, their high performance was achieved by limiting the number of factors take into consideration. In addition, often, integrating the results obtained from multiple studies might not be so straightforward, due to the heterogeneity of the methods employed and the requirement for an independent external validation. Indeed, the need for validating the models on external sets of samples is an aspect that requires care. One main limitation to a wider application of the phenolic profiles might also be the need of sufficient sample sizes that are able to sufficiently represent the high number of (known and unknown) study variables at play and their interaction. Finally, a comprehensive view could be achieved only when the full relationships between the phenolic profile and the variables is more deeply understood.

## Figures and Tables

**Table 1:** Chemical markers proposed to differentiate wines produced with different grape varieties.

Grape Variety	Type of Wine	Chemical Markers	Role of Chemical Markers	Analytical Method	Statistical Method	References
Aurelius, Chardonnay, Müller Thurgau, Moravian Muscat, Hibernal, Malverina, Merzling	W	*p*-coutaric acid, caftaric acid, protocatechuic and syringic acid; (+)-catechin, (−)-epicatechin	Different set of specific phenolic acids (absolute concentrations)	HPLC-DAD	CVA	[42]
Chardonnay, Riesling, Sauvignon Blanc	W	Shikimic acid, ferulic acid, *trans*-caffeic acid, epicatechin	The most significant loadings in the NMR spectrum	NMR	LDA	[43]
Chardonnay, Muscat, Cabernet Sauvignon, Cienna, Dolcetto, Durif, Merlot, Petit Verdot, Pinot Noir, Primitivo, Syrah, Zinfandel	W, R	Caffeic acid, galic acid, rutin, *trans*-resveratrol	Different content of caffeic acid, gallic acid and rutin in wine samples; lack of *trans*-resveratrol in Chardonnay and Muscat	HPTLC	HCA, SVD, PCA, ANN	[10]
Müller-Thurgau, Riesling	W	Amino and organic acids, phenolic acids, flavonoids and stilbenes	High concentration of quercetin, kaempferol, resveratrol in Müller-Thurgau; high (+)-catechin, (−)-epicatechin, caftarate and coutarate in Riesling	NMR	PLS	[44]
Lemberger, Pinot Blanc, Pinot Gris, Müller-Thurgau, Riesling, Gewürztraminer, Pinot Noir	W, R	Phenolic compounds (quercetin, catechin, resveratrol, gallate)	Specific fingerprints due to variety, origin, vintage, physiological state, and technological treatment	NMR	CV, LDA, MANOVA, MC, NCM, PCA,	[45]
Passerina, Verdicchio	W	Various phenolic compounds	High concentration of tyrosol, quercetin and glucuronide in Passerina; and hydroxytyrosol, caffeic, caftaric, coumaric and 2-S-glutathionylcaftaric acids (including their esters) in Verdicchio	HPLC–DAD	PCA	[46]
Bianco d’Alessano, Chardonnay, Falanghina, Fiano, Malvasia, Moscato, Negroamaro, Verdecca, Malvasia Nera, Primitivo, Susumaniello	W, Rs, R	Gallic acid, syringic acid, luteolin, quercitin, hydroxytyrosol, *trans*-resveratrol	Specific set of absolute concentrations of phenolics	HPLC-DAD	GDA, PCA	[23]
Blaufränkisch, Blauer Portugieser, Pinot Noir, Sankt Laurent, Zweigeltrebe	Rs	Caftaric acid, coumaric acid, ferrulic acid, catechin, malvidin-3-*O*-glucoside, epicatechin, *cis*- and *trans*-resveratrol	Specific set of absolute concentrations of phenolics	HPLC-DAD	Box and Whisker Plot	[37]
Cabernet Sauvignon, Feteasca Neagra, Mamaia, Merlot, Pinot Noir	R	Anthocyanins, anthocyanins ratios, phenolic and	Different contents (expressed in malvidin-3-*O*-glucoside) of acylated malvidin and acylated malvidin/malvidin in combination with acids in different grape varieties	HPLC-PDA, NMR	LDA, PCA	[58]
Aspiran, Bonarda, Cabernet Sauvignon, Malbec, Merlot, Sangiovese, Syrah, Tempranillo	R	Malvidin-3-*O*-glucoside, malvidin-3-(6-*O*-acetylglucoside), malvidin-3-*O*-glucoside-4-vinylguaiacol	Absolute concentration (not specified according grape variety)	HPLC-MS	MCR-ALS, D-UPLS	[14]
Aglianico (Ag), Cannonau (Ca), Corvina, Montepulciano, Nebbiolo (Nb), Nerello (Nr), Primitivo (Pr), Raboso, Sagrantino, Sangiovese (Sa), Teroldego (Te)	R	Hydroxycinnamates (Ca), Anthocyanins (Te), flavan-3-ols (Ag, Sa, Nb, Nr), flavonols (Sa), amino acids (Pr)	Specific classes of markers through grape varieties	UHPLC-QTOF MS	ANOVA, PCA	[56]
Corvina Veronese, Negroamaro, Primitivo, Raboso Piave	R	Ratios of anthocyanins, various phenolic compounds, volatile profile	High concentration of disubstituted compounds and lower acyl derivatives in Corvina and Negroamaro; high content of trisubstituted flavonoids in Primitivo; and anthocyanic and non-anthocyanic acyl derivatives in Raboso	UHPLC-Q/TOF	HCA, PCA, Tukey test	[59]
Amarone, Recioto, Primitivo	R	Ratios laricitrin, delphinidin, and petunidin	Ratios have identified the use of 10% Primitivo in wine blends	UHPLC-Q/TOF	HCA, Heat maps, Tukey test	[60]
Aspiran, Bonarda, Cabernet Sauvignon, Malbec, Merlot, Sangiovese, Syrah, Tempranillo	R	Phenolic acids and anthocyanins	Differentiation of Malbec; less effective for geographical origin	HPLC-DAD	MCR–ALS	[61]
Baboso, Castellana, Listán Negro, Listán Prieto, Merlot, Negramoll, Ruby Cabernet, Syrah, Tintilla, Vijariego.	R	Flavonols and anthocyanins	Lower or higher absolute concentrations	HPLC-DAD	ANOVA, PCA, Pearson coefficient	[18]
Bonarda, Cabernet Sauvignon, Malbec, Merlot, Syrah, Tempranillo	R	Phenolic acids, flavan-3-ols, anthocyanins	Specific set of the absolute concentrations of phenolics	HPLC-MS	ANOVA, HSD	[62]
Cabernet Sauvignon, Graciano, Tempranillo	R	Flavan-3-ols	Absolute concentrations of phenolics	HPLC-DAD/ESI-MS	-	[63]
Cabernet Sauvignon, Pinotage, Syrah	R	Flavan-3-ols and phenolic acids	Specific set of the absolute concentrations of phenolics	HPLC-DAD	Multiple linear regression analysis	[64]
Cabernet Sauvignon, Cabernet Franc, Carménère, Merlot, Syrah	R	Flavan-3-ols and phenolic acids	Absolute concentrations of phenolics	HPLC-DAD/PDA	PCA	[65]
Cabernet Franc, Merlot, Sangiovese, Syrah	R	Flavan-3-ols, tannins	Lower or higher absolute concentrations	HPLC-DAD-MS	ANOVA, PCA, Tukey test	[64]
Carménère, Merlot	R	Ratio of total quercetin/total myricetin and concentration of myricetin	Different ratio contents	HPLC-DAD-ESI-MS^n^	DA, PCA	[29]
Chardonnay, Graševina, Malvazija Istarska, Maraština Muscat Blanc; Pošip, Cabernet Sauvignon, Merlot, Plavac mali, Teran	W, R	Phenolic acids, flavonoids, tannins and stilbenes	Specific concentrations for each wine; the content of *cis*-piceid—discriminant for white wines, peonidin 3-(6″-acetyl)-glucoside and taxifolin—for red wines	UHPLC-QqQ- MS/MS	ANOVA, LSA, SLDA	[19]
Rías Baixas, Ribeira Sacra	R	Syringic acid, malvin, oenin, (+)-catechin, (−)-epicatechin, quercetin, *trans*-resveratrol	Specific absolute concentrations; high amounts of *trans*-resveratrol in Ribeira Sacra and malvin in Rías Baixas	HPLC-DAD	LDA, PCA, SIMCA, SVM	[38]
Vranac, Kratošija, Cabernet Sauvignon	R	Phenolic acids, flavonoids, tannins and stilbenes	Vranac—high content of anthocyanins; Kratošija—high content of hydroxycinnamic acids; Cabernet Sauvignon—high content of flavan-3-ols and low content of stilbenes	HPLC-DAD	ANOVA, LSD	[67]
Negroamaro, Primitivo	R	Gallic acid, syringic acid, catechin, quercetin, hydroxytyrosol, *trans*-resveratrol	Significant differences of amounts of syringic acid and hydroxytyrosol in Primitivo, *trans*-resveratrol in Negroamaro	HPLC-DAD	MVA, PCA, OPLS-DA, SIMCA	[39]
Lambrusco Sorbara, Lambrusco Salamino di Santa Croce, Lambrusco Grasparossa di Castelvetro	R	Caffeic acid, galic acid, *p*-coumaric acid, syringic acid, catechin, miricetin, quercitin	*p*-coumaric and caffeic acids describes Sorbara; gallic acid—Salamino; myricetin and quercitin—Grasparossa	HPLC-DAD	PCA, PLS	[68]
Vilana, Dafni, Kotsifali, Mandilari	W, R	Spectral regions from 1800 to 1500 cm^−1^ and from 1300 to 900 cm^−1^	Different fingerprints (band intensity)	FT-IR	LDA	[32]
Cabernet Sauvignon, Feteasca Neagra, Mamaia, Merlot, Pinot Noir	R	Phenolics compounds in the 250–600 nm region	Different fingerprints (band intensity)	UV-Vis	LDA, PCA, PLS-DA	[1]
Chardonnay, Feteasca Regala, Sauvignon Blanc	W	Signals at 1245, 1575 and 1581 cm^−1^	Different fingerprints (band intensity)	SERS	LDA	[69]
Feteasca Regala, Sauvignon Blanc	W	Mainly phenolic acids at −767, −543, −530, −653, 1608 and −881 cm^−1^	Different fingerprints (band intensity)	FT-Raman	SLDA	[70]
Sangiovese, Nebbiolo, Aglianico, Nerello Mascalese, Primitivo, Raboso, Cannonau, Teroldego, Sagrantino, Montepulciano, Corvina	R	Tannins	Different fingerprints (band intensity)	MIR	LDA, PCA, SIMCA, SVM	[9]
Chardonnay, Pinot Gris, Riesling, Sauvignon	W	Flavonoids, tannins, stilbenes	Different fingerprints (band intensity)	FT-Raman	LDA	[71]
Chardonnay, Gewürztraminer, Sauvignon Blanc, Lagrein, Cabernet Franc, Cabernet Sauvignon, Merlot, Pinot Nero	W, R	Ratios of cyclic prodelphinidins and the sum of cyclic and non-cyclic prodelphinidins	High or low ratios according to variety	HPLC-DAD-HRMS/MS	PCA	[72]
Chardonnay, Gewürztraminer, Sauvignon Blanc, Lagrein, Cabernet Franc, Cabernet Sauvignon, Merlot, Pinot Nero	W, R	Ratios of cyclic procyanidins and the sum of cyclic and non-cyclic procyanidins	High or low ratios according to variety	HPLC-DAD-HRMS/MS	PCA	[73]

ANN—artificial neural networks; ANOVA—analysis of variance; CV—cross-validation; CVA—canonical variate analysis; D-UPLS—discriminant unfolded partial least-squares; DA—discriminant analysis; DAD—diode array detection; FT-IR—Fourier-transform infrared spectroscopy; GDA—geometric data analysis; HCA—hierarchical cluster analysis; HPLC—high-performance liquid chromatography; HPTLC—High-performance thin-layer chromatography; HSD—honestly significant difference; IR—infrared spectroscopy; LDA—linear discriminant analysis; LSD—least significant difference; MANOVA—multivariate analysis of variance; MC—Monte Carlo resampling approach; MCR-ALS—multivariate curve resolution alternating least square; MIR—mid-infrared spectroscopy; MS—mass spectrometry; MVA—multivariate analysis; NCM—nearest class mean; NMR—nuclear magnetic resonance; OPLS—orthogonal partial least squares; PCA—principal component analysis; PDA—photometric diode array; PLS—partial least squares; QqQ—triple quadrupole; R—red wines; Rs—rosé wine; SERS—surface-enhanced Raman spectroscopy; SIMCA—soft independent modelling by class analogy; SLDA—soft linear discriminant analysis; SVD—singular value decomposition; SVM—support vector machine; TOF—time-of-flight; UHPLC—ultra high-performance liquid chromatography; UV-Vis—ultraviolet–visible spectroscopy; W—white wine; -: No statistical analyses were used.

**Table 2:** Anthocyanins proposed to differentiate wines produced with different grape varieties of *Vitis vinifera*, measured by LC.

Grape Variety	Chemical Markers and Their Role	References
Tempranillo	Mv-3-glu > Pt-3-glu > Dp-3-glu > Pn-3-glu > Cy-3-glu, Mv-3-ace > Pt-3-ace > Pn-3-ace > Dp-3-ace > Cy-3-ace, *trans*-Mv-3-cum > Dp-3-cum > Pn-3-cum > *cis*-Mv-3-cum > Cy-3-cum, Mv-caf > Pn-caf	[22]
Tortosi	All present, except: Cy-3-glu, Pt-3-ace, Pn-3-ace, Dp-3-cum, Pn-caf	
Bobal	Low concentrations of anthocyanins, Pt-3-ace, Pn-3-ace and Pn-caf were not present	
Moravia Agria	Cy-3-ace, Mv-3-ace, *cis*-Mv-3-cum, Pn-3-cum, *trans*-Mv-3-cum and Mv-caf only	
Moravia Dulce	Dp-3-glu, Pt-3-glu, Pn-3-glu, Mv-3-glu, Cy-3-ace, Mv-3-ace, *cis*-Mv-3-cum, Pn-3-cum, *trans*-Mv-3-cum only	
Rojal	Pn-3-glu, Mv-3-glu, Cy-3-ace only	
Cabernet Sauvignon	Mv-3-glu, Mv-3-ace, Mv-3-cum, Dp-3-glu, Pt-3-glu and Pn-3-glu	[27]
Garnacha	Highest concentration of Mv-3-glu and low concentrations of other anthocyanins	
Graciano	Mv-3-glu > Pn-3-ace > Mv-3-cum > Pt-3-glu > Dp-3-glu	
Merlot	Mv-3-glu > Mv-3-ace > Pn-3-glu > Mv-3-cum > Dp-3-glu > Pt-3-glu	
Tempranillo	Higher content of 3-O-glucosides (except Mv-3-glu) than Cabernet Sauvignon	
Syrah	Pn-3-glu > Pt-3-glu > Dp-3-glu	[50]
Tannat	Pt-3-glu > Dp-3-glu > Pn-3-glu	
Alicante Bouschet, Cabernet Sauvignon, Merlot, Monastrel Bouschet	Various concentrations of Dp-3-ace, Cy-3-ace and Pt-3-ace; Pn-3-glu is a major anthocyanin only for Bouschet varieties	[51]
Bobal, Garnacha, Petit Bouschet, Tempranillo	Not present: Dp-3-ace, Cy-3-ace and Pt-3-ace; Pn-3-glu is a major anthocyanin	
Cabernet Sauvignon, Lingua di Femmina, Merlot, Piedirosso	Specific absolute concentrations distribution: Mv-3-glu > Mv-3-ace > Mv-3-cum	[52]
Aglianico del Taburno, Aglianico del Vulture, Aglianico di Taurasi	Specific absolute concentrations distribution: Mv-3-glu > Mv-3-cum > Mv-3-ace	
Blaufränkisch, Blauer Portugieser, Saint Laurent	Specific set of absolute concentrations: Dp-3-glu, Mv-3-glu, Dp-3-ace for each variety	[53]
Nebbiolo	Mv-3-glu > Pn-3-glu (Pn-3-glu is half of Mv-3-glu concentration)	[56]

LC—liquid chromatography; Cyanidin-3-acetyl-glucoside (Cy-3-ace), Cyanidin-3-*p*-coumaryl-glucoside (Cy-3-cum), Cyanidin-3-*O*-glucoside (Cy-3-glu), Delphinidin-3-acetyl-glucoside (Dp-3-ace), Delphinidin-3-*p*-coumaryl-glucoside (Dp-3-cum), Delphinidin-3-*O*-glucoside (Dp-3-glu), Malvidin-3-acetyl-glucoside (Mv-3-ace), *cis*-Malvidin-3-*p*-coumaryl-glucoside (*cis*-Mv-3-cum), *trans*-malvidin-3-*p*-coumaryl-glucoside (*trans*-Mv-3-cum), Malvidin-3-*O*-glucoside (Mv-3-glu), Malvidin-caffeoyl-3-glucoside (Mv-caf), Peonidin-3-acetyl-glucoside (Pn-3-ace), Peonidin-caffeoyl-3-glucoside (Pn-caf), Peonidin-3-*p*-coumaryl-glucoside (Pn-3-cum), Peonidin-3-*O*-glucoside (Pn-3-glu), Petunidin-3-acetyl-glucoside (Pt-3-ace), Petunidin-3-*p*-coumaryl-glucoside (Pt-3-cum), Petunidin-3-*O*-glucoside (Pt-3-glu).

**Table 3:** Chemical markers for the wine authenticity and quality control in terms of non-*Vitis vinifera* grape species or hybrid species.

Grape Species	Type of Wine	Chemical Markers	Role of Chemical Marker	Analytical Method	Statistical Method	References
Hybrid grape Isabel (*Vitis vinifera* × *Vitis labrusca*)	R	3,5-diglucosidic anthocyanins	The presence in the hybrid grape wine	HPLC-DAD-ESI-MS^n^	-	[81]
Hybrid grape Rondo (*Vitis amurensis* × *Vitis vinifera)*	R	3,5-diglucosidic anthocyanins, gallic acid, *trans*-piceid and *cis*-piceid	High concentrations in Rondo	UPLC-PDA-MS/MS	HCA	[5]
Spine grape (*Vitis davidii Foex*)	R, W	Malvidin-3,5-diglucoside, syringetin-3-*O*-glucoside, dihydroquercetin-3-hexoside and coutaric acid	High concentrations in Spine grapes wine	HPLC-DAD/ESI-MS	ANOVA, Duncan’s multiple range tests	[82]
*Vitis amurensis*, *Vitis davidii*	R	3,5-diglucosidic anthocyanins, phenolic acids, kaempferol-3-*O*-glucoside, quercetin-3-*O*-rhamnoside	Higher concentrations in *Vitis davidii*	HPLC-QqQ-MS/MS	PCA	[83]
Hybrid grapes: Baco (*V. Vinifera* × *V. Labrusca* × *V. Riparia* × *V. Rupestris* × *V. aestivalis*), Seybel (*V. Vinifera* × *V. Rupestris* × *V. lincecu*), Clinton (*V. Labrusca* × *V. riparia*), Jacquez (*V. Aestivalis* × *V. Cinerea* × *V. Vinifera*), Othello (*V. Labrusca × V. Riparia × V. Vinifera*)	R	Proportion of mono-glucoside and acetylated anthocyanins	The ratio lower than three indicates hybrid grape wine	LC-MS-MS	-	[84]
Beihong (*Vitis vinifera* × *Vitis amurensis*)	R	Mainly phenolic and amino acids	Specific fingerprints	NMR	PCA	[85]

-: No statistical analysis were used.

**Table 4:** Phenolics as chemical markers proposed for the geographical origin.

Grape Variety	Wine Origin	Chemical Markers	Role of Chemical Markers	Analytical Method	Statistical Method	References
Riesling	Czech Republic	Gallic acid, caffeic acid, caftartic acid, *p*-coutaric acid, ferulic acid ethylester, *p*-coumaric acid ethylester, (+)-catechin, (−)-epicatechin	Different absolute concentrations of phenolics in comparison with Riesling from other origins from the literature	HPLC-DAD	ANOVA, CVA, LSD	[87]
Riesling	Czech Republic	Protocatechuic acid, *p*-hydroxybenzoic acid, caftaric acid, *p*-coutaric acid, *trans*-resveratrol, *cis*-resveratrol	Specific absolute concentration of each phenolic through five regions	HPLC-DAD	ANOVA, CDA, LSD, PCA	[13]
Riesling	Czech Republic	*p*-coutaric acid, *trans*-resveratrol, *cis*-resveratrol, (+)-catechin, (−)-epicatechin	Specific absolute concentration of each phenolic through five regions	HPLC-DAD	ANOVA, CVA, LSD, PCA	[8]
Aurelius, Chardonnay, Müller Thurgau, Moravian Muscat, Hibernal, Malverina, Merzling	Czech Republic	Protocatechuic acid, *p*-hydroxybenzoic acid, caftaric acid, *cis*-piceid, (+)-catechin and (–)-epicatechin	Specific set of absolute concentrations of each phenolic through two regions	HPLC-DAD	ANOVA	[40]
Chardonnay, Feteasca Regala, Sauvignon Blanc	France and Romania	Mainly phenolic acids at 655, 703, 755, 834, 973, and 1601 cm^−1^	Different fingerprints (band intensity)	SERS	LDA	[69]
Feteasca Regala, Sauvignon Blanc	France and Romania	Mainly phenolic acids at −709, −887, −740, −721, −503, and −628 cm^−1^	Different fingerprints (band intensity)	FT-Raman	SLDA	[70]
Chardonnay, Pinot Gris, Riesling, Sauvignon	France and Romania	Mainly phenolic acids at −451, 1453, −455, 503, 1407, 1428, and 1457 cm^−1^	Different fingerprints (band intensity)	FT-Raman	LDA	[81]
Chardonnay, Pinot Gris, Riesling, Sauvignon Blanc	France and Romania	Gallic acid, ferulic acid, *cis*-caftaric acid, quercitin	Different fingerprints (peak intensity)	NMR	LDA	[43]
Chardonnay, Cabernet Sauvignon	Australia	Cinnamic acid, tartaric acid, myricetin	Specific absolute concentrations (not presented in the article)	HPLC-DAD/MS	LDA, PCA	[89]
Chardonnay	Australia, France, Israel, Italy	Mainly flavonols, flavan-3-ols, flavones, flavanones	Specific absolute concentrations for each region and country	UHPLC-ESI/QTOF-MS	ANOVA, OPLS-DA	[88]
Bobal, Cabernet Sauvignon, Garnacha, Merlot, Tempranillo	France and Spain	Flavonols	Specific molar percentage of eight flavonols through the samples	LC-DAD-ESI-MS^n^	Student-Newman-Keuls test	[28]
Cabernet Sauvignon, Cabernet Franc, Carménère Merlot, Syrah	China	Flavan-3-ols and phenolic acids	Significant differences in the absolute concentrations	HPLC-DAD/PDA	PCA	[66]
Cabernet Sauvignon	China	Gallic acid, (+)-catechin, (−)-epicatechin, procyanidin B1, procyanidin B2, procyanidin C1	Specific absolute concentrations	HPLC-QqQ-MS/MS	PCA, PLS-DA, OPLS-DA	[12]
Cabernet Sauvignon	Bulgaria, Croatia, Macedonia, Montenegro, Serbia	Catechin, quercetin	Specific absolute concentrations through the samples from different countries	HPLC-DAD/FL	ANOVA	[91]
Syrah	Brazil	Catechin	Notable differences in absolute concentrations through the *terroirs*	HPLC-DAD	ANOVA, PCA, Tukey test	[11]
Merlot	Argentina, Brazil, Chile, Uruguay	Anthocyanins	Specific absolute concentration	HPLC-DAD/MS	SVM	[6]
Cabernet Sauvignon, Merlot	China	Percentage of malvidin-3-*O*-glucoside and its derivatives to the total content of anthocyanins	Specific percentage through the *terroirs*	HPLC-MS/MS	Tukey test	[92]
Teran, Plavac mali, Merlot, Cabernet Sauvignon	Croatia	Anthocyanins	Specific absolute concentrations	UPLC-QqQ-MS/MS	ANOVA, LSA, SLDA	[19]
Aspiran, Bonarda, Cabernet Sauvignon, Malbec, Merlot, Sangiovese, Syrah, Tempranillo	Argentina	Malvidin-3-*O*-glucoside and its derivatives	Specific absolute concentrations through the *terroirs* (not presented in the article)	HPLC-MS	MCR-ALS, D-UPLS	[14]
Cabernet Sauvignon	Bulgaria, Croatia, Macedonia, Montenegro, Serbia	Anthocyanins	Specific absolute concentrations	HPLC-DAD, DPPH	-	[93]
Blaufränkisch, Blauer Portugieser, Saint Laurent	Czech Republic	Delphinidin-3-*O*-glucoside, malvidin-3-*O*-glucoside, delphinidin-3-*O*-*p*-coumarylglucoside	Different absolute concentrations of anthocyanins in comparison with studied wines from other origins from the literature	HPLC-DAD	ANOVA, CDA, LSD, PCA	[53]
Various monovarietal and blended wines	Spain	Hydroxycinnamic acids (for white wines), anthocyanin (for red wines)	Not specified	UV-Vis	SVM	[94]
Malbec	Argentina	(+)-Catechin, caftaric acid and quercetin-3-*O*-glucoside	Specific absolute concentrations through six regions (lowest content was in Rivadavia)	HPLC-DAD	DA	[15]
Rías Baixas, Ribeira Sacra	Spain	Syringic acid, malvin, oenin, (+)-catechin, (−)-epicatechin, quercetin, *trans*-resveratrol	Different absolute concentrations (not presented in the article)	HPLC-DAD	LDA, PCA, SIMCA, SVM	[35]
Lemberger, Pinot Blanc, Pinot Gris, Müller-Thurgau, Riesling, Gewürztraminer, Pinot Noir	Germany	Phenolic and amino acids	Specific fingerprint	NMR	CV, LDA, MANOVA, MC, NCM, PCA,	[43]
Moschofilero, Asyrtiko, Agiorgitiko, Mandilaria,	Greece	Gallic acid, *trans*-caffeic, (−)-epicatechin	Both W and R wines from Santorini had twice more of gallic acid and lower concentrations of *trans*-caffeic, (−)-epicatechin than wines from Nemea	NMR	*t* test	[7]

CDA—categorical data analysis; DPPH—2,2,1-diphenyl-1-picrylhydrazyl radical scavenging; ESI—electrospray ionization;. FL—fluorescence.

**Table 5:** Chemical markers for the winemaking quality control.

Grape Variety	Winemaking Method	Chemical Markers	Role of Chemical Markers	Analytical Method	Statistical Method	References
Not reported	Cava sparkling wine	Phenolics with absorbance at 280 nm	Decrease of hydroxycinnamic acids in sparkling wine	HPLC-DAD/ESI–TOFMS	MANOVA	[96]
Chardonnay (Cd), Macabeu, Xarel·lo, Parellada, Pinot Noir (PN), Garnacha (Gn), Trepat (Tp)	Cava sparkling wine	Phenolic acids	High amounts Cd, PN, Gn and Tp low—in Macabeu, Xarel·lo and Parellada	HPLC-DAD	PCA	[97]
Pinot Blanc	Prefermentative cold maceration with pectolytic enzyme	*Trans*-caftaric acid and astilbin	Higher concentrations in wines with cold maceration	HPLC-DAD/FLD, HPLC-MS	PCA	[98]
Primitivo	Destemmed 100%, 75%, 50% of grapes cluster and stem contact for all time fermentation	Anthocyanins	Concentration increasing when less stems are used	HPLC-DAD	ANOVA, Duncan multiple comparison test, PCA	[99]
Merlot, Syrah, Tannat	Traditional maceration; addition of maceration enzymes; cold soak	Anthocyanins	Higher concentration during cold soak	HPLC-DAD	Tukey test	[50]
Pinot Noir	Thermal maceration; treatment after a stuck fermentation; fermentation with 20% whole grape clusters; 100% raisin grapes	Ratios of cyclic and non-cyclic proanthocyanidins	Higher concentration of proanthocyanidins with raisin grapes	HPLC-HRMS/MS	PCA	[100]
Cabernet Sauvignon, Merlot, Monastrell	Wine blending	Groups of phenolics at 520 nm and 620 nm, petunidin-3-*O*-glucoside and peonidin-3-*O*-glucoside	Specific fingerprints through different blends	HPLC-DAD, UV-Vis spectrometry	DA	[101]

**Table 6:** Phenolics as chemical markers for aging in wood.

Grape Variety	Aging Time	Aging Method	Chemical Markers	Role of Chemical Markers	Analytical Method	Statistical Method	References
Chardonnay, Pinot Gris, Verdicchio, Amarone, Sagrantino, Sangiovese, Tannat	1986–2016	In bottles	Sulfonated indoles for white wines, sulfonated, monomeric, and oligomeric flavan-3-ols for red wines	Specific absolute concentrations through different varieties and vintages	UHPLC-MS/MS	One-way ANOVA, Tukey test	[104]
Blend of Cabernet Sauvignon and Merlot	200 days	Medium toasted and highly toasted barrels from oak	Ellagic acid	Higher concentration in wine from oak barrels	HPLC-DAD	-	[103]
Syrah	2, 4, 6, 9, and 12 months	Toasted acacia and French oak barrels	2,4-dihydroxybenzoic acid and flavonoids	Present only in wine aged in acacia wood	LC–DAD/ESI-MS	-	[105]
Tempranillo	3 and 6 months in barrels; 25 days with chips	Non-toasted and in medium toasted chestnut barrels and chestnut chips	Benzoic acids, anthocyanins	Specific absolute concentrations of increasing on the aging and distributing differently due to aging method	LC–DAD/ESI-MS	PCA	[106]
Blend of Sangiovese and Merlot	4 months	Oak and cherry wood barriques, steel tanks	Flavanones	Present only in cherry barriques	HPLC-DAD	Post-hoc, LSD, PCA	[107]
Tinta del País	6 and 12 months	Traditional barrels, oak chips and oak staves (American, French and Hungarian), stainless-steel tanks	Epicatechin, phenolic acids, anthocyanins	Specific absolute concentrations of phenolics influenced by aging time, type and wood	HPLC-DAD	LDA, PCA	[108]
Mencia, Tinta del País	3, 6, 9, and 12 months	Oak barrels and oak chips (American and French)	Anthocyanins	Specific set of absolute concentrations (not presented in the article)	HPLC	DA	[110]
Aglianico, Montepulciano	12 months	With and without oak chips	Anthocyanins, tannins	Polymerization of these markers when oak chips are used	HPLC–DAD/ ESI–MS/MS	*t* test, PCA	[110]
Vilana, Dafni, Kotsifali, Mandilari	3, 6, 9, and 12 months	Medium toasted barrels (French oak, American oak, acacia and chestnut), stainless steel	Spectral regions from 1800 to 1500 cm^−1^ and from 1300 to 900 cm^−1^	Different fingerprints according to aging time and type	FT-IR	LDA	[111]
Vilana, Dafni, Kotsifali, Mandilari	3, 6, and 9 months	Tanks with oak sticks and barrels (French oak, American oak, acacia, and chestnut)	Ellagitannins	Content decrement: chestnut > French oak > American oak > chips > acacia	FT-IR	PLS	[32]

**Table 7:** Chemical markers proposed to determine the vintage year.

Grape Variety	Vintage	Chemical Markers	Role of Chemical Markers	Analytical Method	Statistical Method	References
Müller-Thurgau, Riesling	2006, 2007	Amino, organic and phenolic acids	High concentration of different acids through vintages	NMR	PLS	[44]
Moschofilero, Asyrtiko, Agiorgitiko, Mandilaria,	2005, 2006	Gallic acid, *trans*-caffeic acid, *p*-coumaric acid, syringic acid, ferulic acid, (+)-catechin, (−)-epicatechin, quercetin, kaempferol, *trans*-resveratrol	Lower concentration of polyphenols of samples from 2005	NMR	*t* test	[7]
Lemberger, Pinot Blanc, Pinot Gris, Müller-Thurgau, Riesling, Gewürztraminer, Pinot Noir	2008, 2009	Phenolic and amino acids	Individual fingerprint of samples	NMR	CV, LDA, MANOVA, MC, NCM, PCA,	[45]
Chardonnay, Feteasca Regala, Sauvignon Blanc	2011–2015	Malic and tartaric acids	Different fingerprints (band intensity)	SERS	LDA	[69]
Feteasca Regala, Sauvignon Blanc	2011–2015	Mainly phenolic acids at −767, −543, −530, −653, 1608 and −881 cm^−1^	Different fingerprints (band intensity)	FT-Raman	SLDA	[70]
Chardonnay, Pinot Gris, Riesling, Sauvignon	2012–2016	Caffeic, caftaric, ferulic acids	Different fingerprints (band intensity)	FT-Raman	LDA	[71]
Pinotage	1996–2002	Caffeic acid, malvidin-3-*O*-glucoside (MvGl), pinotin A	Increased ratio of caffeic acid/MvGl through time and pinotin A content	HPLC-MD	SD	[113]
Tempranillo	2000–2002	Delphinidin-3-*O*-glucoside (DpGl), petunidin-3-*O*-glucoside (PtGl), glucoside, malvidin-3-*O*-*p*-coumarylglucoside (MvGlCm), malvidin-3-*O*-glucoside (MvGl)	Increment of DpGl, PtGl and malvidin- MvGlCm and decrement of MvGl through time	HPLC	ANOVA, HCA, PCA	[114]
Sangiovese	2008–2010	Anthocyanins	Decrement of anthocyanins and increment of pinotin A through time	UHPLC-DAD-MS/MS	PLS	[115]
Cabernet Sauvignon (CS), Merlot (M)	Range 1978–2005 (for CS) and 1979–2003 (for M)	Anthocyanins, tannins	The specific sum of concentrations of phenolic classes through the samples	HPLC–DAD	ANOVA, PCA	[17]
Varietal red wines	2000–2010	Monomeric anthocyanins, malvidin-3-*O*-glucoside; pyranoanthocyanins are not effective	Decrement of monomeric anthocyanins	LC-ESI-MS	t-test, PCA	[116]
Cabernet Franc (CF), Merlot (M), Sangiovese (Sg), Syrah (Sr)	2006, 2007	Flavan-3-ols, tannins	Lower or higher concentrations (2006 < 2007 in CF, M; 2006 > 2007 in Sg, Sr)	HPLC-DAD-MS	ANOVA, PCA, Tukey test	[65]
Cabernet Sauvignon	1971–2003	Phenolic acids, flavonoids and resveratrol	Decrement and increment of concentrations of specific phenolics during the timeline	HPLC-MS	PCA, PLSR	[112]
Cabernet Sauvignon	2003–2015	(+)-catechin, (−)-epicatechin, malvidin-3-*O*-glucoside, malvidin- 3-*O*-acetylglucoside	Specific set of absolute concentrations (not presented in the article)	HPLC-QqQ-MS/MS	PCA, PLS-DA, OPLS-DA	[12]
Cabernet Sauvignon, Feteasca Neagra, Mamaia, Merlot, Pinot Noir	2009–2017	Phenolics and other chemical compounds in the 1600–900 cm^−1^ spectral region	Different fingerprints	FT-IR	LDA, PCA, PLS-DA	[1]
Cabernet Sauvignon, Feteasca Neagra, Mamaia, Merlot, Pinot Noir	2009–2014	Mainly delphinidin-3-*O*-glucoside, peonidin-3-*O*-glucoside, malvidin-3-*O*-acetylglucoside, malvidin-3-*O*-*p*-coumarylglucoside, peonidin-3-*O*-(6-*p*-coumaroyl)glucoside	Specific set of concentrations and ratios of anthocyanins (expressed in mg/L of malvidin-3-*O*-glucoside) together with NMR fingerprint	HPLC-PDA, NMR	LDA, PCA	[58]

MD—multidimensional; PLSR—partial least squares regression; SD—standard deviation.
